# Reporting of drug induced depression and fatal and non-fatal suicidal behaviour in the UK from 1998 to 2011

**DOI:** 10.1186/2050-6511-15-54

**Published:** 2014-09-30

**Authors:** Kyla H Thomas, Richard M Martin, John Potokar, Munir Pirmohamed, David Gunnell

**Affiliations:** 1 School of Social and Community Medicine, University of Bristol, Canynge Hall 39 Whatley Road, Bristol BS8 2PS, UK; 2 The Academic Unit of Psychiatry, University of Bristol Bristol, UK; 3 Centre for Drug Safety Science, University of Liverpool Liverpool, UK; 4 Health and Wellbeing Division, Department for Children, Adults and Health, South Gloucestershire Council Badminton Road, Yate, Bristol, UK

**Keywords:** Adverse drug reaction, Suicide, Non-fatal suicidal behaviour, Self injury, Depression, Yellow card, Adverse effects

## Abstract

**Background:**

Psychiatric adverse drug reactions (ADRs) are distressing for patients and have important public health implications. We identified the drugs with the most frequent spontaneous reports of depression, and fatal and non-fatal suicidal behaviour to the UK’s Yellow Card Scheme from 1998 to 2011.

**Methods:**

We obtained Yellow Card data from the Medicines and Healthcare products Regulatory Agency for the drugs with the most frequent spontaneous reports of depression and suicidal behaviour from 1964 onwards. Prescribing data were obtained from the NHS Information Centre and the Department of Health. We examined the frequency of reports for drugs and estimated rates of reporting of psychiatric ADRs using prescribing data as proxy denominators from 1998 to 2011, as prescribing data were not available prior to 1998.

**Results:**

There were 110 different drugs with ≥ 20 reports of depression, 58 with ≥10 reports of non-fatal suicidal behaviour and 33 with ≥5 reports of fatal suicidal behaviour in the time period. The top five drugs with the most frequent reports of depression were the smoking cessation medicines varenicline and bupropion, followed by paroxetine (a selective serotonin reuptake inhibitor), isotretinoin (used in acne treatment) and rimonabant (a weight loss drug). Selective serotonin reuptake inhibitors, varenicline and the antipsychotic medicine clozapine were included in the top five medicines with the most frequent reports of fatal and non-fatal suicidal behaviour. Medicines with the highest reliably measured reporting rates of psychiatric ADRs per million prescriptions dispensed in the community included rimonabant, isotretinoin, mefloquine (an antimalarial), varenicline and bupropion. Robust denominators for community prescribing were not available for two drugs with five or more suicide reports, efavirenz (an antiretroviral medicine) and clozapine.

**Conclusions:**

Depression and suicide-related ADRs are reported for many nervous system and non-nervous system drugs. As spontaneous reports cannot be used to determine causality between the drug and the ADR, psychiatric ADRs which can cause significant public alarm should be specifically assessed and reported in all randomised controlled trials.

## Background

Adverse drug reactions (ADRs) cost the UK’s NHS up to £2 billion each year [1]. In recent years there has been growing concern that certain prescribed medicines may be associated with psychiatric adverse drug reactions such as depression, non-fatal self-harm and suicide [2,3]. The occurrence of medication induced suicide is particularly distressing to the general public. In the UK, television programmes such as the British Broadcasting Company (BBC) programme “Secrets of Seroxat” which was first aired in October 2002 and “Dying for clear skin” shown in November 2012, have attracted record viewing figures and public response [4,5]. These documentaries focussed on the possible risk of suicide with the antidepressant paroxetine and isotretinoin (used to treat severe acne) and showed that drug- induced psychiatric ADRs have the potential to cause significant public alarm. This may lead to adverse health outcomes if unfounded safety concerns result in the reduced use of effective medicines. When a drug is first licensed for use in the general population, there is limited information about its possible adverse effects, as pre-marketing drug trials are underpowered to detect rare psychiatric ADRs such as suicide [6]. Therefore post marketing surveillance using spontaneous reporting systems is crucial, particularly for rare outcomes. However, only a small number of studies have systematically described the medicines which are associated with spontaneous reports of psychiatric ADRs [7-10]; to the best of our knowledge, this has never been done before in the UK.

The aim of this paper is to identify the drugs with the most frequent reporting of suspected psychiatric ADRs to the UK’s Yellow Card Scheme from 1998 to 2011. We focus on depression and fatal and non-fatal suicidal behaviour. Although drug induced suicide is the psychiatric ADR that is most likely to cause significant public concern, we also include reports of depressive illness and non-fatal suicidal behaviour which are known to be important risk factors for completed suicide [11].

## Methods

### Yellow card data

The Yellow Card Scheme is used by the Medicines and Healthcare products Regulatory Agency (MHRA) to monitor the safety of currently licensed medicines and vaccines in the UK and is part of routine pharmacovigilance. Currently, health professionals (doctors, dentists, nurses, and pharmacists), coroners, patients, parents and carers are encouraged to report ‘suspected’ adverse drug reactions to the scheme using paper Yellow Cards or electronic reports (https://YellowCard.mhra.gov.uk accessed 27th February 2014).

We received preliminary data from the MHRA on all spontaneous reports to the Yellow Card Scheme from its creation in 1964 until the 25^th^ January 2012 using the following Higher Level Terms (HLTs) from the Medical Dictionary for Regulatory Affairs (MedDRA): (a) Depressive disorders; and (b) Suicidal and self injurious behaviour. Details of the Preferred Terms (PTs) which are included in the HLTs are shown in Additional file 1. Due to the large number of drugs involved (Yellow Card reports of depressive disorders were received for 872 medicines, reports of non-fatal suicidal behaviour were received for 425 medicines and reports of fatal suicidal behaviour were received for 196 medicines) we requested detailed individual reports for drugs using the following pragmatically selected thresholds:

1. Twenty or more reports for depressive disorders with a non-fatal outcome.

2. Ten or more reports for suicidal and self injurious behaviour with a non-fatal outcome

3. Five or more reports for suicidal and self injurious behaviour with a fatal outcome

For reports of suicidal and self injurious behaviour associated with a fatal outcome (i.e. completed suicide), the MHRA also provided us with information regarding whether the medicine had also been taken in overdose; this is important as in some cases suicide may have been the result of the deliberate ingestion of excessive quantities of medicines (e.g. self poisoning with pain killers such as paracetamol and co-proxamol) and not actually an adverse effect of the drug. Additionally, new European Union (EU) legislation has amended the definition of the term adverse reaction to include unintended effects from unauthorised as well as authorised use of medication [12]. Age- and sex- specific data were provided in an aggregated format and were not available for individual reports. Data were anonymised, so the suspected ADR could not be linked to either the patient or the reporter. We obtained permission for use of Yellow Card data from the Independent Scientific Advisory Committee for MHRA database research. Ethics approval was not required for this study as Category 1b data are releaseable under the Freedom of Information Act (FOIA) 2000.

### Prescribing data

Prescription cost analysis (PCA) data provide details of the number of items and the net ingredient cost of all prescriptions which have been dispensed in the community in England. Yearly prescription cost analysis data for England from 1998 to 2011 were obtained from the NHS Information Centre (http://www.hscic.gov.uk/prescribing accessed 13th September 2012) and used as crude proxy denominators for the yearly use of individual drugs. Individual drug data on the number of prescriptions dispensed were not available prior to 1998.

For drugs that were not commonly prescribed in the community (we defined this as <100 000 prescriptions in the 14 year study period) we also obtained data from 2008–2011 on hospital usage of medicines by acute trusts from the Commercial Medicines Unit of the Department of Health (cmu.dh.gov.uk). These data provided an estimation of yearly usage for those medicines that are more likely to be prescribed in hospitals than by general practitioners (GPs) in the community. Hospital usage data were not available prior to 2008.

### Classification of medicines

We used the 2014 Anatomical Therapeutic Chemical (ATC) classification system to categorise the medicines [13]. Medicines are classified based on their main indication for use worldwide and there is only one ATC code for each route of administration.

We stratified the medicines into nervous system medications and non-nervous system medications according to their ATC classification to assess whether our psychiatric outcomes of interest were more likely to be reported for nervous system drugs than drugs which affect other systems. Although bupropion is only licensed for use as a smoking cessation medicine in the UK, its ATC code was changed in 2009 from ATC level 5 N07BA02 (under ATC level 4 N07BA- Drugs used in nicotine dependence) to N06AX12 (under ATC level 4 NO6AX-Other antidepressants) to represent its main indication worldwide (personal communication WHO Collaborating Centre for Drug Statistics Methodology).

### Statistical analyses

Stata version 12.0 (StataCorp, USA) and Excel 2007 (Microsoft, USA) were used for the analyses. We produced frequency tables for the drugs (all, nervous system and non nervous system drugs) with the most frequent reports of depression and non-fatal and fatal suicidal behaviour from 1998 to 2011. For drugs with reports of fatal suicidal behaviour we also examined the percentage of these reports that included ingestion of the specific medicine in an overdose (i.e. self-poisoning with the medicine).

We calculated overall rates of reporting of suspected ADRs per million prescriptions from 1998 to 2011 using the number of reports for each drug as the numerator and the number of prescriptions for each drug from the PCA data as the denominator [14]. Scatter plots of the reporting rates of depressive disorders versus non-fatal suicidal behaviour and versus suicide were created and Spearman’s correlation coefficients were calculated to examine possible correlations between the rates of reporting of depression with non-fatal and fatal suicidal behaviour. We used Spearman’s correlation coefficient as it is a non parametric method which does not assume that the data are normally distributed; this is particularly relevant in small samples.

## Results

### Psychiatric ADR reporting 1964–2012

Over the entire time period (1964 to 25^th^ January 2012) there were 6800 Yellow Card reports (35.8% male) of depressive disorders, 3624 reports (42.5% male) of non-fatal suicidal behaviour and 664 reports (69.6% male) of suicide; these reports were 1.65% of all reports made to the database. The ratio of reports of non-fatal to fatal suicidal behaviour was 5.5: 1. Eighteen to 35 year olds accounted for 24.6% of reports of depressive disorders, 28.9% of reports of non-fatal suicidal behaviour and 29.7% of suicide reports. People aged 36 to 65 years accounted for 47.7% of reports of depressive disorders, 43.8% of reports of non-fatal suicidal behaviour and 48.2% of suicide reports. Ten percent of reports of depressive disorders, 4.2% of non-fatal suicidal behaviour reports and 7.1% of suicide reports were from people aged ≥ 66 years.

### Reports and prescribing 1998–2011

Using our thresholds, there were 110 different drugs with 20 or more Yellow Card reports of depressive disorders, 58 with 10 or more reports of non-fatal suicidal behaviour and 33 with five or more reports of suicide from January 1^st^ 1998 to December 31^st^ 2011.

**Figure 1:**
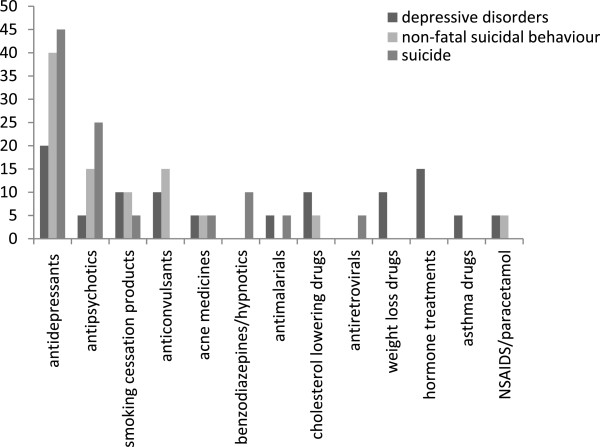
Distribution of major classes of the Top 20 medicines with the highest numbers of Yellow Card reports for depressive disorders and fatal and non-fatal suicidal behaviour.

For all drugs, the top five medicines for reports of depressive disorders were the smoking cessation medicines, varenicline (1^st^) and bupropion (2^nd^), paroxetine, a selective serotonin reuptake inhibitor (SSRI) antidepressant (3^rd^), isotretinoin, used in acne treatment (4^th^) and rimonabant, a weight loss drug (5^th^). The top five medicines for reports of non-fatal suicidal behaviour were paroxetine (1st), varenicline (2nd), the SSRI antidepressants citalopram (3rd) and fluoxetine (4th) and clozapine, an antipsychotic (5th). With the exception of varenicline and clozapine all of the drugs with the highest reporting of non-fatal suicidal behaviour are SSRIs. The drug with the most frequent reports of suicide was clozapine, an antipsychotic agent (1^st^), followed by three SSRIs, citalopram (2^nd^), fluoxetine (3^rd^) and paroxetine (4^th^) and a serotonin noradrenaline reuptake inhibitor (SNRI), venlafaxine (5^th^). Although bupropion and isotretinoin were in the top five for reports of depressive disorders, for reports of non- fatal suicidal behaviour, bupropion was ranked 7^th^ and isotretinoin was ranked 11^th^. For suicide, bupropion was ranked 22^nd^ and isotretinoin was ranked 7^th^. Although varenicline was ranked 1^st^ for reports of depressive disorders and second for reports of non-fatal suicidal behaviour, for suicide it was ranked 6^th^. Unlike the other smoking cessation drugs, there were only 17 Yellow Card reports of depressive disorders, five reports of non-fatal suicidal behaviour and no reports of suicide for nicotine replacement therapy.Figure 1 shows the distribution of the major drug classes of the top 20 medicines with the greatest number of Yellow Card reports of depressive disorders, non-fatal suicidal behaviour and suicide. Reports of psychiatric adverse reactions were most frequently observed for antidepressants (20% of drugs in the top 20 for reports of depressive disorders, 40% for reports of non-fatal suicidal behaviour and 45% for suicide reports). Antipsychotic agents were also commonly implicated (25% of suicide reports and 15% of reports of non-fatal suicidal behaviour) as well as anticonvulsants (10% of reports of depressive disorders and 15% of reports of non-fatal suicidal behaviour). Other drug classes such as smoking cessation drugs, weight loss medicines and antimalarials also had frequent reports of psychiatric ADRs (smoking cessation medicines- 10% of reports of depressive disorders and non-fatal suicidal behaviour, 5% of suicide reports; weight loss medicines- 10% of reports of depressive disorders; and antimalarials- 5% of reports of depressive disorders and suicide).

With the exception of varenicline, bupropion and paroxetine, most of the top 10 drugs with reports of depressive disorders are treatments for non nervous system indications such as isotretinoin (an anti-acne preparation), rimonabant (an anti-obesity preparation), simvastatin (a lipid modifying agent), mefloquine (an antimalarial), levonorgestrel (a hormone), atorvastatin (a lipid modifying agent) and rofecoxib (an anti-inflammatory and antirheumatic product). However for reports of non-fatal and fatal suicidal behaviour, most of the top 10 drugs were nervous system drugs such as antidepressants, antipsychotics and the smoking cessation medicines.

The drugs (nervous system drugs versus non nervous system drugs) with reports of more than one psychiatric ADR stratified by ATC level 1 classification are listed in Additional file 2. Table 1 shows the percentage of Yellow Card reports of suicide which involved intentional overdose (i.e. deliberate ingestion of the medicines). Drugs that were commonly implicated in fatal overdose were aspirin, where 100% of suicide reports involved deliberate ingestion of the drug, followed by 66.7% for tramadol, a narcotic analgesic, 61.5% for zopiclone, a hypnotic medicine and 60% for paracetamol (an analgesic).

**Table 1:** Percentage of Yellow Card reports for suicides where the implicated drug was taken in an episode of fatal self-poisoning from 1998 to 2011

**Drug**	**Number of suicide reports**	**Percentage of overdose in suicide reports from 1998-2011**
Aspirin	4	100.0
Tramadol	6	66.7
Zopiclone	13	61.5
Paracetamol	5	60.0
Diazepam	11	54.5
Amitryptiline	6	50.0
Temazepam	4	50.0
Co-proxamol	4	50.0
Quetiapine	13	46.2
Olanzapine	24	37.5
Venlafaxine	42	31.0
Sertraline	21	19.0
Citalopram	70	17.1
Bupropion*	6	16.7
Clozapine	78	12.8
Mirtazapine	17	11.8
Paroxetine	50	10.0
Varenicline	41	7.3
Escitalopram	15	6.7
Fluoxetine	58	5.2
Risperidone	24	4.2
Duloxetine	28	3.6
Isotretinoin	32	3.1
Aripiprazole	15	0.0
Mefloquine	8	0.0
Efavirenz	7	0.0
Infliximab	5	0.0
Flupenthixol	4	0.0

*Bupropion is licensed for use as an antidepressant in other countries but not the UK.

### Rates of ADRs 1998–2011

In order to account for how often the drug is prescribed we describe reporting rates using community prescribing data next (Table 2). Although the SSRI antidepressants (paroxetine, fluoxetine and citalopram) had the most frequent reports of depressive disorders and fatal and non-fatal suicidal behaviour, reporting rates were low as these drugs are widely prescribed. Additionally, simvastatin had a much lower reporting rate of depressive disorders than mefloquine (0.4 per million prescriptions versus 457 per million) although there was a similar number of reports (110 versus 105 respectively).

**Table 2:** Rates of Yellow Card adverse reports per million prescriptions dispensed for the top 20 drugs with the highest number of adverse reports for depressive disorders, non-fatal suicidal behaviour and suicide

	**Drug**	**Number of reports**	**Overall rate per million prescriptions 1998-2011**	**95% confidence intervals**
*Depressive disorders*				
Non-nervous system drugs	Rimonabant	190	773	667-891
	Isotretinoin	199	553	479-636
	Mefloquine	105	457	373-553
	Etonogestrel	51	82	61-108
	Sibutramine	55	25	19-33
	Levonorgestrel	86	14	12-18
	Desogestrel	64	9	7-12
	Rofecoxib	73	9	7-12
	Montelukast	45	5	3-6
	Atorvastatin	74	0.6	0.5-0.8
	Simvastatin	110	0.4	0.3-0.5
Nervous system drugs	Clozapine	50	630	468-831
	Bupropion*	483	330	301-361
	Varenicline	975	248	233-264
	Topiramate	61	18	14-23
	Levetiracetam	66	15	12-19
	Paroxetine	263	8	7-9
	Venlafaxine	57	2	2-3
	Fluoxetine	56	0.9	0.7-1.2
	Citalopram	56	0.7	0.5-0.9
*Non-fatal suicidal behaviour*				
Non-nervous system drugs	Rimonabant	74	30	236-378
	Isotretinoin	71	197	154-249
Nervous system drugs	Clozapine	132	1664	1393-1973
	Atomoxetine	126	230	192-274
	Varenicline	675	172	159-185
	Bupropion*	117	80	66-96
	Duloxetine	97	32	26-39
	Paroxetine	709	21	19-22
	Levetiracetam	69	16	12-20
	Topiramate	37	11	8-15
	Pregabalin	47	6	5-8
	Escitalopram	61	6	4-7
	Venlafaxine	113	4	3-5
	Risperidone	49	3	3-5
	Mirtazapine	67	3	3-4
	Olanzapine	48	3	2-4
	Citalopram	219	3	2-3
	Fluoxetine	161	3	2-3
	Sertraline	51	2	1-3
	Paracetamol	69	0.4	0.3-0.5
*Suicide*				
Non-nervous system drugs	Efavirenz	7	2312	930-4757
	Isotretinoin	32	89	61-126
	Mefloquine	8	35	15-69
Nervous system drugs	Clozapine	78	984	778-1227
	Varenicline	41	10	8-14
	Duloxetine	28	9	6-13
	Aripiprazole	15	9	5-14
	Risperidone	24	2	1-3
	Paroxetine	50	2	1-2
	Venlafaxine	42	1	1-2
	Olanzapine	24	1	0.9-2
	Escitalopram	15	1	0.8-2
	Quetiapine	13	1	0.6-2
	Fluoxetine	58	0.9	0.7-1
	Citalopram	70	0.8	0.7-1
	Mirtazapine	17	0.8	0.5-1
	Sertraline	21	0.8	0.5-1
	Zopiclone	13	0.2	0.1-0.4
	Diazepam	11	0.2	0.1-0.3
	Amitryptiline	6	0.1	0.03-0.2

*Bupropion is licensed for use as an antidepressant in other countries but not the UK.

For medicines in the top 20 that were more commonly prescribed in secondary care such as efavirenz and clozapine, rates were re-calculated using hospital prescribing data for the most recent years. The rate of reporting for efavirenz was 27.4 per million hospital prescriptions for suicide (compared to 2312 per million community prescriptions) which would move efavirenz from 1^st^ to 3^rd^ place for non nervous system drugs associated with suicide (see Table 2). Clozapine had a reporting rate of 19.7 per million hospital prescriptions for depressive disorders (630 per million community prescriptions), 62.3 per million hospital prescriptions for non-fatal suicidal behaviour (1664 per million community prescriptions) and 36.3 per million hospital prescriptions for suicide (984 per million community prescriptions). Therefore clozapine remained at the top of the rankings for nervous system drugs with the highest reporting rate of suicide.

**Figure 2:**
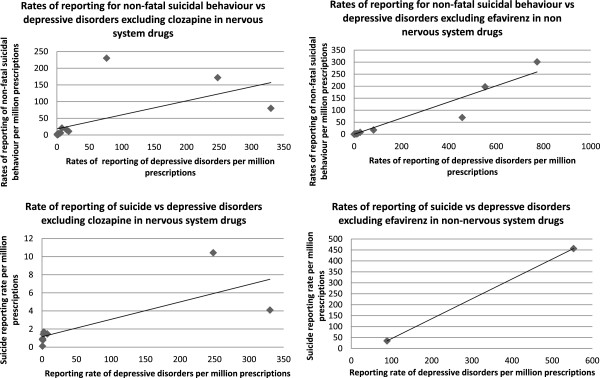
Scatter plots of reporting rates of suicide versus rates of depressive disorders and rates of non-fatal suicidal behaviour versus rates of depressive disorders for nervous system medicines (excluding clozapine) and non nervous system medicines (excluding efavirenz).

Figure 2 shows scatter plots of the reporting rates of non-fatal suicidal behaviour versus depressive disorders and suicide versus depressive disorders for nervous system versus non nervous system drugs (see Additional file 2). Clozapine and efavirenz were excluded as these drugs are not commonly prescribed in the community. For nervous system drugs there was positive correlation between reporting rates of non-fatal suicidal behaviour and depressive disorders (n = 14, Spearman’s rho 0.88, p < 0.001) as well as for suicide and depressive disorders (n = 9 Spearman’s rho 0.88, p < 0.01). For non- nervous system drugs there was slightly weaker positive correlation between the rates for non-fatal suicidal behaviour and depression (n = 11, Spearman’s rho 0.78, p < 0.01). Reports of depressive disorders and suicide were observed for only two non nervous system drugs so Spearman’s rho was not calculated.

## Discussion

### Summary of findings

Reports of drug induced depression and fatal and non-fatal suicidal behaviour constitute a very small minority of total ADR reports. We found that the drugs with the highest frequency of Yellow Card reports of psychiatric ADRs included nervous system medicines such as the SSRI antidepressants, smoking cessation medicines varenicline and bupropion and the antipsychotic drug clozapine as well as non-nervous system drugs such as isotretinoin (used in acne treatment) and mefloquine (an antimalarial). Reports of depression were most frequently observed for non nervous system drugs; most reports of suicide and non-fatal suicidal behaviour involved nervous system medicines. Analgesics (such as paracetamol) and hypnotics/benzodiazepines were also likely to be implicated in reports of suicide but most of these deaths were reported to have been caused by deliberate ingestion of excessive quantities of the drugs in overdose. Reporting rates for efavirenz and clozapine were much lower when data on hospital usage were used instead of community prescriptions as these drugs are not likely to be prescribed in a primary care setting. There was positive association between the reporting rates of self-harm and depression and suicide and depression for nervous system and non-nervous system drugs.

### Strengths and limitations

This is the first study to systematically describe the reporting of depression and fatal and non-fatal suicidal behaviour to the UK’s Yellow Card Scheme although it excludes other psychiatric adverse drug reactions, such as anxiety, psychosis and insomnia. Strengths include the 14 years of reporting and the inclusion of drug utilisation data (prescribing data) to calculate reporting rates. We examined the reports from all medicines that met pre-defined thresholds for inclusion and stratified the medicines by their ATC level 1 classification. The occurrence of Yellow Card reports of suspected ADRs with a specific drug does not indicate that the drug definitely caused the ADR as there are many factors that have to be taken into consideration when assessing causal relationships, such as the timing of when the drug was taken in relation to the outcome, the biological plausibility, the possible contribution of concomitant medication and the underlying disease [15].

Additionally, the reporting of ADRs using Yellow Cards is affected by the extent of use of a particular drug, whether the drug is newly licensed (i.e. length of time of drug on the market) [16], stimulated reporting caused by safety warnings about a drug (notoriety bias) [17], adverse media publicity about a drug and the seriousness of the ADR [18]. In our study there were 5.5 times as many non-fatal suicidal behaviour as suicide reports, whereas the ratio of non-fatal self-harm to suicide in the general population is 30:1[19]. This is likely to have occurred because more serious (or fatal) adverse reactions are reported more often. Under-reporting is a serious issue; only 6% of ADRs in hospital and general practice are reported in the UK [20,21]. Also, drugs such as oral contraceptives and beta blockers which have long been recognised as associated with depression, were likely to have low numbers of reports in our study period, as Yellow Card reporting decreases the longer the drug has been available on the market [18]. Over the counter medicines, such as nicotine replacement therapy, may also be subject to under-reporting.

Due to the large numbers of drugs with reports of our psychiatric ADRs of interest, we used specific thresholds to request detailed individual reports. Although this approach is likely to have identified those medicines which would have potentially caused the most harm, it may have missed drugs which are rarely prescribed, but which have a high ratio of reports to prescriptions and drugs which have only recently been marketed with low consumption.

The number of Yellow Card reports received cannot be used to determine the actual incidence rate of an ADR as both the total number of reactions occurring and the number of patients who actually used the drug are unknown (although we used community prescribing data as a proxy). We used PCA data for the number of prescriptions dispensed in the community to estimate a rate of ADR reports per million prescriptions of the drug; this is a very crude estimation and did not include standardisation by age or sex or take account of the average duration of prescribing or the indication for which the drug was prescribed. Age and sex standardisation are important as most non-fatal suicidal behaviour occurs in women and at younger ages when general drug prescribing is rarer [22]. The importance of using proxy denominators to estimate reporting rates was shown by the stark contrast in the reporting rates of depression for mefloquine and simvastatin, although similar numbers of absolute reports were obtained for both medicines.

Certain drugs, such as efavirenz and clozapine are more often prescribed in hospitals, which resulted in under-estimation of the denominator and over-estimation of reporting rates in our analyses using community prescriptions. Reporting rates were much lower when hospital prescribing data were used although these data were only available for a more limited time period. Additionally, clozapine is subject to extensive monitoring for adverse effects such as agranulocytosis; this would also increase the reporting of other unrelated adverse effects. Therefore the reporting pattern is likely to be very different for clozapine compared with other medicines for which reporting is truly spontaneous; limiting inference about possible causal associations.

Disproportionality analysis is another method that is often used to identify potential safety hazards or safety signals in spontaneous reporting data. Dal Pan et al. (2013) defined disproportionality as “the finding that a given adverse event/adverse drug reaction is reported for a particular drug more often than would be expected based on the number of reports of that adverse event/adverse drug reaction for all other drugs in the database” [23]. This method has several advantages over the use of proxy denominator data to calculate reporting rates because it is not affected by changes in the reporting of ADRs and does not require the use of external data sources [23]. Statistical methods for determining disproportionality include the use of the proportional reporting ratio (PRR), which can be interpreted in a similar manner to a risk ratio (i.e. a higher ratio indicates a stronger signal) [24]. However, the aim of this study was to provide a descriptive overview of the drugs associated with frequent Yellow card reports of depressive disorders and fatal and non-fatal self-harm as opposed to identifying new safety signals.

### Evidence from other studies of worldwide spontaneous reporting systems

Few studies have examined the spontaneous reporting of psychiatric ADRs [7-10]. Robertson and Allison (2009) used the Food and Drug Administration Adverse Event Reporting System (FDA AERS) to examine whether drugs associated with reports of suicidal ideation were also associated with reports of suicide attempts and found that many different classes of drugs were associated with both outcomes [10]. One recent study examined the reporting of psychiatric ADRs in the Swedish paediatric population using the Swedish Drug Information System (SWEDIS) database. In keeping with our analysis, this study found that isotretinoin, antiepileptics, SSRIs and montelukast (used in the treatment of asthma) were frequently associated with serious psychiatric ADRs [7]. Other frequently implicated drugs included vaccines, centrally working sympathomimetics and melatonin. An earlier study looked at reports to Canada’s adverse drug reaction database from 1965 until the early 1990s. Adrenergic drugs (clonidine and methyldopa), beta blockers (propranolol), corticosteroids, benzodiazepines and oral contraceptives were associated with more than 10 reports of depression although none of these drugs featured prominently in our analysis, probably because we focussed on later years and reports of ADRs decrease the longer a particular drug has been on the market [8]. Vilhelmsson et al. (2011) looked at reports of psychiatric ADRs with antidepressant medication that were made to a consumer association in Sweden [9]. Depression was reported as an ADR for citalopram, escitalopram and paroxetine. Suicidal behaviour was reported as a psychiatric ADR for all included antidepressants, mainly SSRIs and serotonin noradrenaline reuptake inhibitors (SNRIs). These findings are also consistent with our analysis. Last, Aagaard and Hansen (2013) examined ADRs reported by consumers in Europe for nervous system medications including antiepileptics, antidepressants and smoking cessation medicines [25]. Most of the ADRs were reported for psychiatric disorders which also accounted for the majority of ADRs categorised as serious [25].

### Other methods used to investigate psychiatric ADRs

There are three main explanations for the higher frequency of reports of depression and suicidal behaviour observed with certain drugs. First, the drug may have a causal role in triggering the adverse drug reaction. Second, the drug may have no causal role but is used in a condition which increases the risk of developing the adverse outcome, for example an association between antidepressants and suicide may be explained by the fact that depression itself is a risk factor for suicide [11]. Third, the drug may or may not have a causal role but there are reasons why patients, healthcare professionals and other individuals are more likely to send in a spontaneous report for the drug. For example, in the UK there was an increase in spontaneous reports of ADRs for paroxetine following adverse media publicity from the BBC Panorama programmes which suggested an association between paroxetine and suicidal behaviour [26]. In addition, the association between isotretinoin and inflammatory bowel disease observed in the FDA AERS has been explained by an excess of lawyer-initiated reports related to pending isotretinoin lawsuits [27]. Although spontaneous reporting systems are important for identifying previously unknown ADRs, RCTs and meta-analyses of RCTs are the preferred study designs for the evaluation of drug therapies, as if properly conducted, they provide the strongest evidence of causality between exposure to a particular drug and the outcome of interest [28]. The strongest evidence of adverse psychiatric ADRs comes from meta-analyses of placebo controlled randomised controlled trials (RCTs). These show increased risk of self-harm and suicidal thinking in children prescribed SSRIs such as paroxetine compared with placebo (3.7% vs 2.5%, RR 1.51:95% CI 0.62 to 3.69) [29]. Such evidence has resulted in regulatory action to restrict the use of SSRIs in young people in many countries. The authorisation for rimonabant was suspended by the European Medicines Agency in October 2008 because its adverse psychiatric effects, particularly depression, were felt to outweigh its benefits as a mildly effective weight loss drug [2]. These findings were also obtained from meta-analyses of randomised controlled trials and are in keeping with our findings of a high reporting rate of depression and non-fatal self-harm with rimonabant [30].

More recently, the MHRA in the UK and their equivalent in the US, the Food and Drug Administration (FDA), have issued warnings in relation to certain drugs such as varenicline, based on spontaneous reports of suspected ADRs in individual cases from the Yellow Card Scheme in the UK and its equivalent in the USA [31]. Prescription event monitoring studies have found conflicting results; a study carried out in New Zealand found that varenicline use was associated with more reports of suicide and suicidal ideation whereas a more recent study in England did not find any evidence for an increase in reports of depression and other neuropsychiatric adverse events with varenicline [32,33]. However, observational cohort studies have not found any definitive evidence that varenicline and bupropion are associated with increased suicidal behaviour [17,34].

In June 2009 the FDA requested the addition of information regarding neuropsychiatric adverse effects to the precautions section of montelukast prescribing information [35]. A review of Merck drug company trials failed to identify any completed suicides associated with its use [36], a finding supported by analysis of a large primary care database in the UK [37]. A meta-analysis of suicidal risk during treatment with clozapine also found a lower overall risk of suicidal behaviour associated with clozapine compared with other treatments for psychosis [38]. Although efavirenz has been associated with spontaneous reports of psychiatric ADRs, there was no clear evidence from a systematic review that patients taking efavirenz were at increased risk of suicide [39]. A recent review found little evidence for an increased risk of suicide and suicidal behaviour with antidepressants, antiepileptics, varenicline, montelukast and antipsychotics [40]. It is reassuring that, with the exception of SSRIs in young people, other study designs have not confirmed elevated risks of neuropsychiatric adverse effects for medicines with high numbers of spontaneous reports in our study.

## Conclusions

Spontaneous Yellow Card reports of depression and suicide-related ADRs were obtained for many different classes of drugs including nervous system drugs such as antidepressants, antipsychotics and smoking cessation medicines, in addition to non- nervous system drugs such as weight loss medicines and drugs used in the treatment of acne. Although we could not examine emerging safety concerns for recently marketed drugs, it is reassuring that no new drugs were identified. As spontaneous reports of ADRs do not indicate causal associations between drugs and adverse reactions, we suggest that psychiatric adverse events which can cause significant public alarm such as suicide, suicidal behaviour and depression should be specifically monitored and reported in all randomised controlled trials.

## Competing interests

All authors have completed the Unified Competing Interest form at http://www.icmje.org/downloads/coi_disclosure.pdf (available on request from the corresponding author) and declare:

KHT has received support from the National Institute for Health Research for the submitted work, has no financial relationships with any organisations that might have an interest in the submitted work in the previous 3 years and has no other relationships or activities that could appear to have influenced the submitted work. RMM has no support for the submitted work, had specified relationship with the MHRA in the previous 3 years (is a member of the MHRA’s Independent Scientific Advisory Committee for CPRD research and receives expenses and a small fee for meeting attendance and preparation for meetings) and has no other relationships or activities that could appear to have influenced the submitted work. DG had no support for the submitted work, had specified relationship with the MHRA in the previous 3 years (is a member of the MHRA’s Pharmacovigilance Expert Advisory Group and receives travel expenses and a small fee for meeting attendance and preparation for meetings) and has no other relationships or activities that could appear to have influenced the submitted work. MP has no support for the submitted work, had specified relationship with the MHRA in the previous 3 years (is Chair of the Pharmacovigilance Expert Advisory Group of the Commission on Human Medicines and receives travel expenses and a small fee for meeting attendance and preparation for meetings) and has no other relationships or activities that could appear to have influenced the submitted work. JP has no support for the submitted work, has no financial relationships with any organisations that might have an interest in the submitted work in the previous 3 years and has no other relationships or activities that could appear to have influenced the submitted work.

## Authors’ contributions

KT, DG and RMM conceived of the study. KT performed all of the statistical analyses. KT wrote the first draft of the manuscript. All authors contributed to the final draft of the manuscript and have read and approved of the final draft.

## Pre-publication history

The pre-publication history for this paper can be accessed here:

http://www.biomedcentral.com/2050-6511/15/54/prepub

## Supplementary Material

Additional file 1 List of Preferred Terms included in the Medical Dictionary for Regulatory Affairs Higher Level Terms (a) Depressive disorders and (b) Suicidal and self injurious behaviour. Click here for file

Additional file 2 List of drugs with the most frequent reports of depressive disorders and fatal and non-fatal suicidal behaviour. Click here for file
