# Supplementary material for: Reporting of drug induced depression and fatal and non-fatal suicidal behaviour in the UK from 1998 to 2011
Source: BMC Pharmacol Toxicol. 2014 Sep 30;15:54. doi: 10.1186/2050-6511-15-54 (PMC4184159; doi:10.1186/2050-6511-15-54)
Supplement: Additional file 1 — List of Preferred Terms included in the Medical Dictionary for Regulatory Affairs Higher Level Terms (a) Depressive disorders and (b) Suicidal and self injurious behaviour. [file 2050-6511-15-54-S1.docx]

**Additional file 1** List of Preferred Terms included in the Medical Dictionary for Regulatory Affairs Higher Level Terms (a) Depressive disorders and (b) Suicidal and self injurious behaviour

| **Higher Level Terms** | **Preferred Terms** |
| --- | --- |
| Suicidal and self injurious behaviour | *Completed suicide* |
|  | *Intentional self injury* |
|  | *Self injurious behaviour* |
|  | *Suicidal behaviour* |
|  | *Suicidal ideation* |
|  | *Suicidal attempt* |
|  |  |
| Depressive disorders | *Agitated depression* |
|  | *Childhood depression* |
|  | *Depression* |
|  | *Depression postoperative* |
|  | *Depression suicidal* |
|  | *Dysthymic disorder* |
|  | *Major depression* |
|  | *Menopausal depression* |
|  | *Post stroke depression* |
|  | *Postpartum depression* |
|  |  |
