# Supplementary material for: Reporting of drug induced depression and fatal and non-fatal suicidal behaviour in the UK from 1998 to 2011
Source: BMC Pharmacol Toxicol. 2014 Sep 30;15:54. doi: 10.1186/2050-6511-15-54 (PMC4184159; doi:10.1186/2050-6511-15-54)
Supplement: Additional file 2 — List of drugs with the most frequent reports of depressive disorders and fatal and non-fatal suicidal behaviour. [file 2050-6511-15-54-S2.docx]

**Additional file 2** List of drugs with the most frequent reports of depressive disorders and fatal and non-fatal suicidal behaviour

| **Adverse Drug Reactions** | **Non nervous system drugs** | **Nervous system drugs** |
| --- | --- | --- |
| Depression, fatal and non-fatal suicidal behaviour | *Efavirenz* | *Bupropion** |
|  | *Isotretinoin* | *Citalopram* |
|  | *Mefloquine* | *Clozapine* |
|  |  | *Fluoxetine* |
|  |  | *Mirtazapine* |
|  |  | *Paroxetine* |
|  |  | *Risperidone* |
|  |  | *Sertraline* |
|  |  | *Tramadol* |
|  |  | *Varenicline* |
|  |  | *Venlafaxine* |
|  |  | *Zopiclone* |
|  |  |  |
| Depression and non-fatal suicidal behaviour only | *Amlodipine* | *Atomoxetine* |
|  | *Atenolol* | *Gabapentin* |
|  | *Atorvastatin* | *Levetiracetam* |
|  | *Desogestrel* | *Pregabalin* |
|  | *Ethinyloestradiol* | *Topiramate* |
|  | *Etonogestrel* |  |
|  | *Ibuprofen* |  |
|  | *Interferon beta* |  |
|  | *Lansoprazole* |  |
|  | *Levonorgestrel* |  |
|  | *Medroxyprogesterone* |  |
|  | *Montelukast* |  |
|  | *Omeprazole* |  |
|  | *Rimonabant* |  |
|  | *Simvastatin* |  |
|  |  |  |
| Depression and fatal suicidal behaviour only | *Efavirenz* | *Bupropion** |
|  | *Isotretinoin* | *Citalopram* |
|  | *Mefloquine* | *Clozapine* |
|  |  | *Fluoxetine* |
|  |  | *Paroxetine* |
|  |  | *Risperidone* |
|  |  | *Sertraline* |
|  |  | *Tramadol* |
|  |  | *Varenicline* |
|  |  | *Venlafaxine* |
|  |  |  |
| Non-fatal suicidal behaviour and suicide only |  | *Amitryptiline* |
|  |  | *Diazepam* |
|  |  | *Duloxetine* |
|  |  | *Escitalopram* |
|  |  | *Flupenthixol* |
|  |  | *Olanzapine* |
|  |  | *Paracetamol* |
|  |  | *Quetiapine* |
|  |  | *Temazepam* |
|  |  |  |
|  |  |  |

*Bupropion is licensed for use as an antidepressant in other countries but not the UK
